# Semi-quantitative CT score reflecting the degree of pulmonary infection as a risk factor of hypokalemia in COVID-19 patients: a cross-sectional study

**DOI:** 10.3389/fmed.2024.1366545

**Published:** 2024-10-21

**Authors:** Ru Li, Baofeng Wu, Xifeng Yang, Botao Liu, Jian Zhang, Mengnan Li, Yi Zhang, Ying Qiao, Yunfeng Liu

**Affiliations:** ^1^Department of Endocrinology, First Hospital of Shanxi Medical University, Taiyuan, China; ^2^First Clinical Medical College, Shanxi Medical University, Taiyuan, China; ^3^Department of Medical Imaging, Shanxi Medical University, Taiyuan, China; ^4^Department of Pharmacology, Shanxi Medical University, Taiyuan, China; ^5^Department of Radiology, First Hospital of Shanxi Medical University, Taiyuan, China

**Keywords:** COVID-19, SARS-CoV-2, hypokalemia, water-electrolyte imbalance, pneumonia

## Abstract

**Background:**

Hypokalemia is a common electrolyte disorder observed in patients afflicted with coronavirus disease 2019 (COVID-19). When COVID-19 is accompanied by pulmonary infection, chest computed tomography (CT) is the preferred diagnostic modality. This study aimed to explore the relationship between CT semi-quantitative score reflecting the degree of pulmonary infection and hypokalemia from COVID-19 patients.

**Methods:**

A single-center, cross-sectional study was conducted to investigate patients diagnosed with COVID-19 between December 2022 and January 2023 who underwent chest CT scans upon admission revealing typical signs. These patients were categorized into two groups based on their blood potassium levels: the normokalemia group and the hypokalemia group. Medical history, symptoms, vital signs, laboratory data, and CT severity score were compared. Binary regression analysis was employed to identify risk factors associated with hypokalemia in COVID-19 patients with pulmonary infection.

**Results:**

A total of 288 COVID-19 patients with pulmonary infection were enrolled in the study, of which 68 (23.6%) patients had hypokalemia. The CT severity score was found to be higher in the hypokalemia group compared to the normokalemia group [4.0 (3.0–5.0) vs. 3.0 (2.0–4.0), *p* = 0.001]. The result of binary logistic regression analysis revealed that after adjusting for sex, vomiting, sodium, and using potassium-excretion diuretics, higher CT severity score was identified as an independent risk factor for hypokalemia (OR = 1.229, 95% CI = 1.077–1.403, *p* = 0.002).

**Conclusion:**

In this cohort of patients, semi-quantitative CT score reflecting the degree of pulmonary infection may serve as a risk factor of hypokalemia in COVID-19 patients.

## 1 Introduction

Coronavirus disease 2019 (COVID-19), caused by severe acute respiratory syndrome coronavirus-2 (SARS-CoV-2), has emerged as a significant global public health threat in this century. It commonly manifests with symptoms such as fever, cough, dyspnea, and other clinical manifestations. Pulmonary infection is also a prevalent clinical manifestation observed in patients with COVID-19 and one of the primary motives for patients seeking medical attention. Chest computed tomography (CT) is an indispensable imaging modality that plays a crucial role in the screening, diagnosis, and management of the disease. In order to standardize the description of the degree of pulmonary infection, varieties of chest CT scoring system have been developed and demonstrated excellent performance in evaluating the clinical severity (1).

Electrolyte imbalances are frequently observed in patients with COVID-19. A meta-analysis reported a prevalence rate of 24.31% for hypokalemia (2), which has been associated with adverse outcomes including prolonged hospitalization, extended stay in the intensive care unit and requiring invasive mechanical ventilation (3, 4). In the context of SARS-CoV-2 infection, hypokalemia is primarily attributed to increased renal excretion, which can be ascribed to hyperactivation of the renin–angiotensin–aldosterone system (RAAS) and tubular damage. Additionally, gastrointestinal losses and anorexia secondary to severe illness contribute partially to this phenomenon. Furthermore, the utilization of diuretics and corticosteroid therapy has been linked to the occurrence of hypokalemia (5). However, the relationship between pulmonary infection and hypokalemia is not well established.

The objective of this study is to investigate the association between semi-quantitative CT score of pulmonary infection and hypokalemia, aiming to elucidate the potential etiology of hypokalemia in patients with COVID-19 combined with pulmonary infection and provide valuable insights for the early recognition and prevention of hypokalemia in other cases of pneumonia.

## 2 Materials and methods

### 2.1 Study design and patients

A single-center, cross-sectional study was conducted at the First Hospital of Shanxi Medical University. The study was approved by the local Ethics Committee (Approval number: 2018K002).

We investigated the medical records of patients admitted to the hospital in December 2022 and January 2023, who were diagnosed with COVID-19 infection through Real Time Polymerase Chain Reaction (RT-PCR) analysis of nasal and pharyngeal swab specimens and who exhibited typical signs of COVID-19 on chest CT upon admission. Patients with incomplete clinical data, readmitted or transferred due to COVID-19 were excluded.

### 2.2 Data collection

We collected the demographic information (age, sex, height, and weight), comorbidities, medication history, clinical symptoms, vital signs, and laboratory values including white blood cell (WBC), hemoglobin, platelet, lymphocyte, neutrophil, alanine aminotransferase (ALT), aspartate aminotransferase (AST), albumin, urea, creatinine, serum potassium, sodium, chloride, procalcitonin (PCT), brain natriuretic peptide (BNP), troponin, prothrombin time (PT), and activated partial prothrombin time (APTT). Additionally available information of thyroid function including free triiodothyronine (FT3), free thyroxine (FT4), and thyroid stimulating hormone (TSH) were also collected.

According to the serum potassium level at admission, the patients were categorized into two groups: normokalemia group (3.5–5.5 mmol/L) and hypokalemia group (<3.5 mmol/L). Patients with hyperkalemia (>5.5 mmol/L) were excluded.

### 2.3 Semi-quantitative of CT score analysis

The image analysis was performed using Thoracic VCAR on AW VolumeShare 7 (General Electric Company, United States). By adjusting the CT values, the entire lung and lung lesions were automatically segmented. Two experienced radiologists independently conducted a comprehensive reviewed all CT images without access to any clinical information. The percentages of involvement in the left and right lungs were separately recorded, with corresponding scores assigned as follows: 0% (0), 1%–10% (1), 11%–19% (2), 20%–29% (3), 30%–39% (4), 40%–49% (5), and ≥50% (6). The severity score was calculated by summing up the scores for both lungs, ranging from 1 to 12. In case of any discrepancies between the radiologists’ assessments, a final decision was made by a third experienced radiologist.

### 2.4 Statistical analysis

The continuous data was expressed as mean (SD) if it followed a normal distribution; otherwise, it was presented as median (p25, p75). The difference between groups was evaluated using unpaired *t*-test or Mann–Whitney test. Categorical variables were described with number (percentage) and analyzed using Chi-squared test. Kruskal–Wallis test was performed to assess the differences in CT severity scores across multiple groups with varying levels of potassium. Risk factors for hypokalemia were evaluated using binary logistic regression analysis. A *p*-value < 0.05 was considered statistically significant. All statistical analyses were conducted using IBM SPSS Statistic 25.

## 3 Results

### 3.1 Patients

**FIGURE 1:**
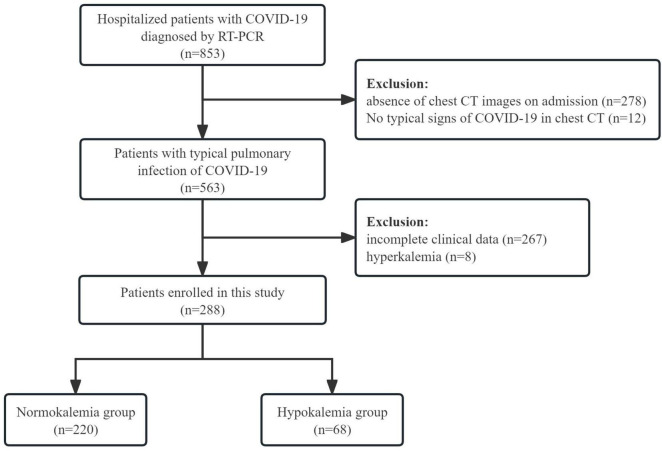
Flowchart of patient screening in this cohort.

A total of 288 COVID-19 patients with pulmonary infection were included in the study (Figure 1), with males accounting for 59% (*n* = 170) of the cohort. The majority of participants were elderly, with a median age of 78 years. Upon admission, the most commonly complained symptoms on admission were fever (76.0%), cough (75.3%), and poor appetite (75.3%). Dyspnea was reported by 44.4% of patients, while myalgia was reported by 13.2%. Vomiting was observed in 9.7% of patients, while diarrhea occurred in 2.4%.

The mean serum potassium level of all patients was calculated to be 3.9 (SD = 0.5) mmol/L. Among the patients, 68 (23.6%) had lower serum potassium levels ranging from 2.21 to 3.49 mmol/L, with a mean value of 3.2 (SD = 0.3) mmol/L. In contrast, 220 (76.4%) patients exhibited normal serum potassium levels, with an average level of 4.1 ± 0.4 mmol/L (Table 1).

**TABLE 1:** Demographic and clinical characteristics of 288 patients classified by serum potassium.

	Overall (*n* = 288)	Normokalemia (*n* = 220, 76.4%)	Hypokalemia (*n* = 68, 23.6%)	*p*-Value
**Demographic information**				
Male	170 (59.0%)	141 (64.1%)	29 (42.6%)	0.002^a^
Female	118 (41.0%)	79 (35.9%)	39 (57.4%)	
Age (years)	78.0 (67.0–84.0)	77.5 (67.0–84.0)	78.5 (68.3–86.0)	0.424^b^
BMI (kg/m^2^)	23.7 (4.3)	23.7 (4.4)	23.7 (3.8)	0.896^c^
**Comorbidities**				
Hypertension	129 (44.8%)	94 (42.7%)	35 (51.5%)	0.205^a^
Diabetes	80 (27.8%)	64 (29.1%)	16 (23.5%)	0.371^a^
Cardiovascular disease	46 (16.0%)	34 (15.5%)	12 (17.6%)	0.666^a^
Cerebrovascular disease	49 (17.0%)	35 (15.9%)	14 (20.6%)	0.369^a^
**Drugs**				
Insulin	31 (10.8%)	25 (11.4%)	6 (8.8%)	0.555^a^
β receptor agonists	5 (1.7%)	3 (1.4%)	2 (2.9%)	0.734^a^
Potassium-excretion diuretics	12 (4.2%)	4 (1.8%)	8 (11.8%)	0.001^a^
Glucocorticoid	18 (6.3%)	13 (5.9%)	5 (7.4%)	0.886^a^
ACEi/ARB	31 (10.8%)	22 (10.0%)	9 (13.2%)	0.452^a^
NSAIDs	129 (44.8%)	94 (42.7%)	35 (51.5%)	0.205^a^
**Symptoms**				
Fever	219 (76.0%)	166 (75.5%)	53 (77.9%)	0.675^a^
Cough	217 (75.3%)	164 (74.5%)	53 (77.9%)	0.570^a^
Dyspnea	128 (44.4%)	101 (45.9%)	27 (39.7%)	0.368^a^
Myalgia	38 (13.2%)	28 (12.7%)	10 (14.7%)	0.673^a^
Poor appetite	217 (75.3%)	157 (71.4%)	60 (88.2%)	0.005^a^
Disorder of consciousness	26 (9.0%)	16 (7.3%)	10 (14.7%)	0.062^a^
Vomiting	28 (9.7%)	13 (5.9%)	15 (22.1%)	0.000^a^
Diarrhea	7 (2.4%)	4 (1.8%)	3 (4.4%)	0.445^a^
**Vital signs**				
Body temperature (°C)	36.5 (36.3–36.9)	36.5 (36.3–36.8)	36.6 (36.3–37.3)	0.375^b^
Pulse (times/min)	84 (15)	84 (15)	83 (16)	0.816^c^
SBP (mmHg)	130 (18)	130 (17)	131 (20)	0.462^c^
DBP (mmHg)	76 (11)	76 (12)	75 (10)	0.739^c^
**Laboratory values**				
BNP (ng/L)	83.4 (34.9–194.8)	76.7 (32.1–190.1)	101.5 (47.5–216.0)	0.211^b^
Troponin (pg/ml)	11.7 (5.8–26.4)	10.5 (5.4–24.6)	14.1 (10.2–26.8)	0.023^b^
WBC (10^9^/L)	5.9 (4.4–8.7)	5.8 (4.3–8.6)	5.9 (4.6–9.6)	0.461^b^
Hemoglobin (10^12^/L)	133.7 (21.1)	134.4 (22.3)	131.5 (16.4)	0.313^c^
Platelet (10^9^/L)	178.5 (133.3–238.0)	180.0 (130.3–236.5)	177.0 (139.3–250.3)	0.483^b^
Lymphocyte (10^9^/L)	0.9 (0.6–1.3)	0.9 (0.6–1.4)	0.8 (0.6–1.2)	0.233^b^
Neutrophil (10^9^/L)	4.3 (2.9–6.8)	4.2 (2.8–6.5)	4.6 (3.0–8.4)	0.349^b^
NLR	4.57 (2.74–8.96)	4.3 (2.6–8.5)	6.3 (3.1–12.7)	0.071^b^
PLR	212.4 (127.3–329.9)	196.2 (125.3–321.7)	248.8 (137.9–360.8)	0.147^b^
ALT (U/L)	23.0 (15.0–36.0)	23.0 (14.0–37.8)	22.0 (18.3–32.0)	0.889^b^
AST (U/L)	32.0 (22.3–49.0)	30.5 (22.0–48.5)	36.0 (24.0–49.0)	0.188^b^
Albumin (g/L)	35.5 (4.7)	35.8 (4.8)	34.5 (4.2)	0.053^c^
Urea (mmol/L)	5.5 (4.1–7.5)	5.7 (4.2–7.9)	4.9 (3.5–6.1)	0.004^b^
Creatinine (μmol/L)	68.0 (57.0–83.8)	69.0 (58.3–84.0)	62.6 (50.0–82.8)	0.024^b^
Potassium (mmol/L)	3.9 (0.5)	4.1 (0.4)	3.2 (0.3)	0.000^c^
Sodium (mmol/L)	135.0 (130.0–139.0)	135.0 (131.3–139.0)	132.0 (123.5–136.8)	0.000^b^
Chlorine (mmol/L)	99.7 (93.8–102.9)	100.6 (95.3–103.6)	96.5 (89.1–101.4)	0.000^b^
PCT (ng/ml)	0.27 (0.17–0.40)	0.26 (0.19–0.38)	0.29 (0.08–0.58)	0.798^b^
PT (s)	13.6 (12.8–14.4)	13.7 (12.8–14.4)	13.2 (12.7–14.3)	0.058^b^
APTT (s)	31.8 (29.6–34.7)	32.0 (29.8–34.6)	31.4 (28.5–35.5)	0.340^b^
**CT severity score**				
CT severity score	3.0 (2.0–4.0)	3.0 (2.0–4.0)	4.0 (3.0–5.0)	0.001^b^

SBP, systolic blood pressure; DBP, diastolic blood pressure; BMI, body mass index; BNP, brain natriuretic peptide; WBC, white blood cell; ALT, alanine aminotransferase; AST, aspartate aminotransferase; PCT: procalcitonin; PT: prothrombin time; APTT, activated partial prothrombin time; ACEi/ARB, angiotensin converting enzyme inhibitors/angiotensin receptor blocker; NSAIDs, nonsteroidal antiinflammatory drugs; NLR, neutrophils-to-lymphocytes ratio; PLR, platelets-to-lymphocytes ratio.

^a^Chi-squared test.

^b^Mann–Whitney test.

^c^Unpaired *t*-test.

### 3.2 Clinical and laboratory analyses of hypokalemia

There was a significant difference in sex distribution between the normokalemia and hypokalemia groups (*p* = 0.002), with a higher proportion of women observed in the hypokalemia group. No significant differences were found in terms of age, comorbidities, vital signs, BMI, and medications except for potassium-excretion diuretics (*p* = 0.001). The hypokalemia group had a higher proportion of patients with poor appetite (*p* = 0.005) and vomiting before admission (*p* = 0.000).

In biochemical examination, the hypokalemic group exhibited significantly higher troponin levels (*p* = 0.023) and lower levels of urea (*p* = 0.004), creatinine (*p* = 0.024), as well as serum electrolytes including potassium (*p* = 0.000), sodium (*p* = 0.000), and chlorine (*p* = 0.000). Furthermore, the presence of hypokalemia was associated with a higher CT severity score (*p* = 0.001).

Regarding inflammatory indicators such as neutrophils-to-lymphocytes ratio (NLR), platelets-to-lymphocytes ratio (PLR), and PCT, although the hypokalemia group showed higher levels, there were no significant differences (*p* = 0.071, *p* = 0.147, *p* = 0.798) (Table 1).

### 3.3 CT severity score and serum potassium

**FIGURE 2:**
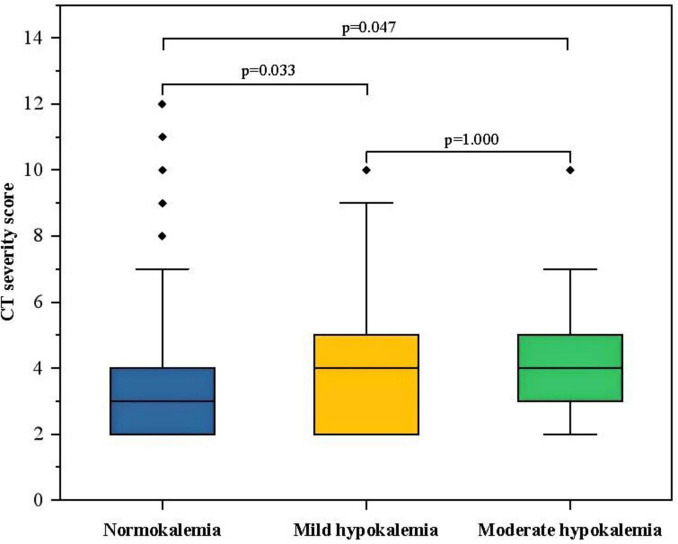
The box-plot of CT severity score in groups with different serum potassium. The CT severity score was 3 (2, 4) in the normokalemia group, 4 (2, 3) in the mild hypokalemia group, and 4 (3, 5) in the moderate hypokalemia group. The Kruskal–Wallis test showed a significant difference between the mild (*p* = 0.033) or moderate (*p* = 0.047) hypokalemia group and the normokalemia group. However, there was no significant difference between moderate and mild hypokalemia groups (*p* = 1.000).

According to the severity of hypokalemia, the hypokalemia group was further categorized into mild hypokalemia (3–3.5 mmol/L) and moderate hypokalemia (<3 mmol/L). Due to a limited number of patients with severe hypokalemia in our cohort, they were not analyzed separately. The results of the Kruskal–Wallis test indicated that both the mild and moderate hypokalemia groups had slightly higher CT severity scores compared to the normokalemia group (*p* < 0.05). However, there was no significant difference between the mild hypokalemia and moderate hypokalemia group (*p* = 1.000) (Figure 2).

The spearman correlation analysis revealed that CT severity score was positively correlated to NLR (*r* = 0.256, *p* < 0.01), PLR (*r* = 0.275, *p* < 0.01), PCT (*r* = 0.230, *p* < 0.01), and negatively correlated with serum potassium level (*r* = −0.153, *p* = 0.01). However, the association between CT severity score and serum potassium disappeared after adjusting for NLR (*r* = −0.095, *p* = 0.108), PLR (*r* = −0.088, *p* = 0.138), and PCT (*r* = −0.109, *p* = 0.064).

### 3.4 Thyroid hormone and serum potassium

To investigate the relationship between thyroid function and hypokalemia, a total of 99 patients were available for analysis. Statistical results showed no significant differences in FT3, FT4, FT3/FT4, and TSH levels between the normokalemia group and the hypokalemia group (Table 2).

**TABLE 2:** Thyroid function in normokalemia group and hypokalemia group.

Thyroid function	Normokalemia (*n* = 71)	Hypokalemia (*n* = 28)	*p*-Value
FT3	3.4 (2.9–4.0)	3.0 (2.5–3.8)	0.145
FT4	16.4 (14.1–19.1)	16.5 (13.2–19.1)	0.920
FT3/FT4	0.22 (0.17–0.25)	0.19 (0.16–0.25)	0.300
TSH	1.8 (1.2–2.9)	1.6 (0.8–3.4)	0.932

FT3, triiodothyronine; FT4, free thyroxine; TSH, thyroid stimulating hormone.

### 3.5 Risk factors for hypokalemia

**FIGURE 3:**
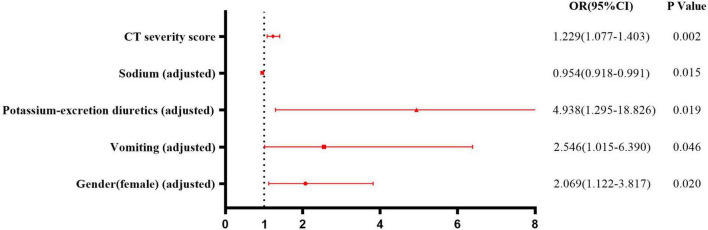
Risk factors for hypokalemia in patients with COVID-19. Binary logistic regression analysis showed after adjusting for sex, vomiting, sodium, and using potassium-excretion diuretics, higher CT severity score was an independent risk factor for hypokalemia (OR = 1.229, 95% CI = 1.077–1.403, *p* = 0.002).

The occurrence of hypokalemia was analyzed as the dependent variable, and variables that showed statistical significance in univariate analysis were included as candidate factors. Variables exhibiting a linear relationship with other factors were excluded. Binary logistic regression analysis (Forward Selection: Likelihood Ratio) showed after adjusting for sex, vomiting, sodium, and the use of potassium-excretion diuretics, a higher CT severity score was an independent risk factor for hypokalemia (OR = 1.229, 95% CI = 1.077–1.403, *p* = 0.002) (Figure 3).

## 4 Discussion

Hypokalemia is considered the second most prevalent electrolyte disorder in pneumonia patients, following hyponatremia. The reported incidence of hypokalemia in community-acquired pneumonia was 15.6% (6). Among hospitalized patients with COVID-19, the proportion can exceed 30% (4, 7). Despite advancements in understanding the mechanisms underlying COVID-19 infection and hypokalemia, limited research has been conducted to investigate the association between pneumonia and hypokalemia.

Although RT-PCR has been widely employed for COVID-19 diagnosis, its results were highly influenced by the duration of symptoms, viral load, and sample quality (8). In a series of 51 patients, chest CT exhibited higher sensitivity compared to RT-PCR (9). Besides, compared with the delay of RT-PCR results, chest CT offered more advantages in the triage of emergency departments (10). Previous studies have proposed multiple quantitative or semi-quantitative chest CT scores for assessing severity of pulmonary infection (1). The CT scores based on the affected area of five lung lobes have been proven to accurately predict the severity of the disease (11, 12). But their correlation with hypokalemia remains unclear. Given our limited inclusion of patients with poor conditions, we modified the scoring criteria in order to better differentiate the severity of pulmonary infections. This innovative finding established the association between CT severity score reflecting pulmonary infection and the risk of hypokalemia.

In this study, a positive association between CT severity score and inflammatory indicators such as NLR, PLR, and PCT was observed. After adjusting for inflammatory indicators such as NLR, PLR, and PCT, the negative association between CT severity score and serum potassium disappeared, suggesting a potential mediation by inflammatory cytokines. Multiple studies have reported a positive correlation between C-reactive protein, erythrocyte sedimentation rate, ferritin, granulocyte/lymphocyte ratio, and CT severity score (13, 14). The excessive release of inflammatory cytokines has been implicated in the rapid progression of the disease. However, there was no significant difference in CT severity score between the moderate hypokalemia group and the mild hypokalemia group. The possible explanation was that more severe cases were not included due to unavailability of chest CT scans upon admission or performance at other medical institutions.

Consistent with our findings, previous studies have also reported a higher susceptibility to hypokalemia in women (7, 15). Nevertheless, the precise mechanism remains elusive. It has been mentioned that estrogen may exert an impact on downregulating the expression of angiotensin-converting enzyme 2 (ACE2) (16). As a reverse regulator of RAAS, downregulated ACE2 promotes the imbalance of the RAAS system, leading to increased potassium exclusion and hypokalemia. However, it is important to note that the majority of women included in our study were postmenopausal patients with significantly reduced estrogen levels. ACE2 also plays a crucial role as a mediator for SARS-CoV-2 virus infection in host cells (17). Normal aging is characterized by increased ACE2 expression and activity predominantly observed in males, thereby rendering them more susceptible to the virus and experiencing enhanced disease severity (18). Considering these factors, the gender disparity observed in ACE2 expression becomes more challenging to explain this phenomenon. Another plausible explanation could be that older women exhibit lower exchangeable potassium levels compared to other populations, as supported by historical evidence (19).

Gastrointestinal manifestations are frequently observed in COVID-19 patients, although the prevalence varies significantly among studies and the relationship between gastrointestinal symptoms and elderly patients remains controversial (20). In our study, poor appetite was reported by 75.3% of patients, vomiting by 9.7%, and diarrhea by only 2.4%. Given the high expression of ACE2 in the gastrointestinal tract (21), it is possible that viruses can disrupt intestinal barrier function through direct cytopathic effects or cytokine release, which may explain these symptoms. Additionally, disturbances to the gut–lung axis and imbalances in gut microbiota have also been implicated (22, 23). We observed significant differences in rates of poor appetite and vomiting between normokalemic and hypokalemic groups but no difference in the incidence of diarrhea. As potassium intake mainly comes from food sources, a decrease in appetite during illness could reduce potassium intake, while vomiting and diarrhea contribute to excess potassium excretion from the digestive tract. However, in our study, the limited prevalence has impeded the investigation of diarrhea as a contributing factor.

Diuretics were widely employed for alleviating fluid load. Potassium-excretion diuretic, such as hydrochlorothiazide and furosemide effectively eliminated water while concurrently expelling a substantial amount of solute, including potassium. Consequently, it is not surprising that prolonged usage of these medications may lead to hypokalemia and other electrolyte imbalances. Consistent with our findings, another study also demonstrated the contributory role of diuretic therapy in COVID-19 patients experiencing hypokalemia. The use of non-steroidal anti-inflammatory drugs (NSAIDs) were frequent during the COVID-19 epidemic due to their potent anti-inflammatory, antipyretic, and analgesic effects. Although medical records indicated that nearly half of the patients had a history of NSAID use, in reality, this proportion was considerably higher. On one hand, NSAIDs inhibit cyclooxygenase enzymes, thereby diminishing renal perfusion and upregulating RAAS through reduced synthesis of prostaglandin E2 and I2 (24). On the other hand, compensatory increase in ACE2 allowed the virus to infect cells more aggressively and increased the susceptibility of the disease. It has also been reported that Ibuprofen inhibited carbonic anhydrase II, leading to renal tubular acidosis and hypokalemia (25, 26). As direct inhibitors of the RAAS pathway, the application of angiotensin converting enzyme inhibitors/angiotensin receptor blocker (ACEi/ARB) further complicated the pathological mechanism of this disease. Although available evidence suggested that their use was not associated with a poor prognosis of disease, changes in serum potassium during infect have not been explored (27). We did not found any differences in NSAIDs and ACEi/ARB usage between groups in the univariate analysis. Therefore, considering their impact on serum potassium levels in the context of COVID-19 requires further investigation.

Thyroid dysfunction may be accompanied by water and electrolyte disorders, which can occur due to direct viral invasion, systemic immune activation, and pituitary dysfunction in affected patients (28). So we attempted to explore the relationship between thyroid hormone and hypokalemia. Our findings revealed no significant differences in FT4 and TSH levels. Although there was a lower trend in FT3 levels in the hypokalemic group, this difference did not reach statistical significance. A retrospective study concluded that electrolyte imbalances were likely only relevant in severe hypo-/hyperthyroidism (29).Thus, thyroid dysfunction was unlikely to be the cause of hypokalemia, at least in our study cohort.

There were some limitations in our study. Firstly, the patients we included were required records of chest CT and complete clinical data upon admission, which constrained our sample size. Moreover, our CT severity score was a semi-quantitative score based only on lesion extent. A quantitative score reflecting the nature and extent of the lesion would provide a more accurate assessment of disease severity (30). More importantly, due to the lack of routine measurement for components of RAAS and urine potassium in clinical settings, it was challenging to directly evaluate their relationship with blood potassium levels. Nevertheless, our findings offered valuable population data regarding the etiology of hypokalemia in COVID-19 patients with pulmonary infection.

## 5 Conclusion

In this study, semi-quantitative CT score reflecting the degree of pulmonary infection was identified as a potential risk factor for hypokalemia in patients with COVID-19. This association may be mediated by inflammatory factors. Therefore, it is imperative to closely monitor the blood potassium levels in patients exhibiting high CT scores. Timely identification and correction of hypokalemia can contribute to effective management and improved prognosis.

## Data Availability

The original contributions presented in this study are included in this article/supplementary material, further inquiries can be directed to the corresponding authors.
